# Efficacy of Ferulic Acid in an Animal Model of Drug-Resistant Epilepsy: Beneficial or Not?

**DOI:** 10.7759/cureus.30892

**Published:** 2022-10-31

**Authors:** Surabhi Thapliyal, Jagjit Singh, Mukesh Mamgain, Ashish Kumar, Manisha Bisht, Ashok Singh, Kiran Meena, Sanjeev Kishore, Shailendra Handu

**Affiliations:** 1 Pharmacology, All India Institute of Medical Sciences, Rishikesh, IND; 2 Medical Oncology, All India Institute of Medical Sciences, Rishikesh, IND; 3 Biochemistry, All India Institute of Medical Sciences, Rishikesh, IND; 4 Pathology, All India Institute of Medical Sciences, Rishikesh, IND

**Keywords:** neuroprotective, matrix mettaloproteinase-9, anti-inflammatory activity, drug-resistant epilepsy, ferulic acid

## Abstract

Background

Lamotrigine (LTG) and subconvulsive doses of pentylenetetrazol (PTZ) as a model mimic drug-resistant epilepsy (DRE), which is a serious unmet medical condition. Previous evidence suggests an imperative role of neuroinflammation in the development of DRE. Various preclinical models of brain injury have reported potent anti-inflammatory and antioxidant properties of ferulic acid (FA). Therefore, its efficacy against intractable epilepsy is worthwhile to study.

Materials and methods

The present study evaluated the efficacy of FA in LTG and PTZ-induced refractory seizures in mice. On every alternate day for 38 days, LTG (5mg/kg) was injected before PTZ (30-40mg/kg) to establish a murine model of DRE. Animals were treated with two doses of FA (40, 80 mg/kg). All the animals were assessed for seizure score and the latency of seizures every alternate day till the end of the study. Histopathological score and the levels of pro-inflammatory mediators, interleukin-1βeta (IL-Iβ), tumor necrosis factor-alpha (TNF-α), and matrix metalloproteinase-9 (MMP-9) were quantified in the brain tissue of these mice.

Results

Ferulic acid (FA) neither decreases the LTG and PTZ-induced refractory seizures score nor increases the latency to develop seizures. In addition, the injury to hippocampal neurons and the levels of pro-inflammatory cytokines were comparable with two doses of FA in treated mice.

Conclusion

In the present study, single-dose FA treatment does not show any beneficial effect against the LTG/PTZ model of DRE. Therefore, its single-dose administration might not be beneficial against the DRE model.

## Introduction

Drug-resistant epilepsy (DRE) is defined as the failure to achieve seizure freedom even after the adequate usage of two anti-epileptic drugs (AEDs) at the right dose and duration [1,2]. The unprovoked recurrent seizure is considered a hallmark of refractory epilepsy and affects almost one-third of the epileptic population [3,4]. Even after numerous AEDs in the market, it is hard to treat DRE [5]. Therefore, poly-therapy is generally advised to treat patients with uncontrollable seizures. However, additive adverse effects could be a major therapeutic challenge with this approach [6].

The precise pathological underlying mechanism behind refractory epilepsy remains obscure. A study on cerebral ischemic rodents reported an association of neuroinflammation with increased expression of the pro-inflammatory cytokines, interleukin-1βeta (IL-1β), and tumor necrosis factor (TNF-α) [7]. That’s why, the levels of pro-inflammatory cytokines such as IL-Iβ, TNF-α, and proteolytic enzymes, matrix metalloproteinase-9 (MMP-9) were found to cause neuronal damage during DRE [8]. In this context, the use of polyphenols has recently drawn attention to the treatment of epilepsy. Besides its antioxidant and anti-inflammatory properties, evidence from various preclinical studies has found ferulic acid (FA) as a remarkable neuroprotectant due to its modulatory actions on multiple neurosignalling pathways [9]. Moreover, it can easily cross the blood-brain barrier (BBB) and provides good bioavailability to treat various neurological disorders [10,11].

The National Institute of Neurological Disorder & Stroke (NINDS) under the Epilepsy Therapy Screening Program provides information for various seizure models to screen promising AEDs. The lamotrigine (LTG)-resistant kindled model has been the most extensively investigated for unraveling the mechanism behind kindling and drug resistance. The kindling process illustrates the roadmap to epileptogenesis that could be achieved by the administration of a subconvulsive dose of a CNS stimulant, such as pentylenetetrazol (PTZ), on every alternate day over a period of time to induce a permanent alteration in the epileptogenic sensitivity of the brain [12]. Thus, the present study was designed to evaluate the efficacy of FA with other AEDs to combat intractable epilepsy in the LTG/PTZ kindling model of mice.

## Materials and methods

Animals

Experiments were performed on 24 adult (25 to 28 g) male Swiss albino mice (received from the Central Animal House facility of All India Institute of Medical Sciences (AIIMS), Rishikesh, India). The mice were kept in 12 h light/dark cycle at a temperature of 23°C ± 2°C, relative humidity of 65%, and were given free access to food and water (ad libitum). The experimental work was duly approved by the Institutional Animal Ethics Committee (protocol approval No. IAEC/AIIMS/Rish/Phar/09/19). Experiments were carried out according to the guidelines laid down by the Committee for the Purpose of Control and Supervision of Experiments on Animals (CPCSEA), Ministry of Fisheries Animal Husbandry & Dairying, Government of India. Moreover, the mice were acclimatized to the laboratory for a week before initiating the experiment. 

Experimental design

The mice were randomized and divided (n=6) evenly into four groups as illustrated in Table 1.

**Table 1:** The four randomized groups for the study PTZ: Pentylenetetrazol, LTG: Lamotrigine, CBZ: Carbamazepine, FA: Ferulic acid, i.p.: Intraperitoneally

Various Groups	Treatment
Group 1: PTZ	No Pretreatment with LTG except for only once on the last day: LTG (15mg/kg, i.p) + CBZ (40mg/kg, i.p.)
Group 2:LTG+PTZ +CBZ	Pretreatment with LTG (5mg/kg, i.p) regularly before PTZ and on the last day LTG (15mg/kg, i.p) + CBZ (40mg/kg, i.p.)
Group 3: LTG+FA (40mg/kg)+PTZ+ CBZ	Pretreatment with LTG (5mg/kg, i.p) regularly before PTZ and on the last day LTG +FA (40mg/kg, p.o) + CBZ
Group 4: LTG+FA (80mg/kg)+PTZ+ CBZ	Pretreatment with LTG (5mg/kg, i.p) regularly before PTZ and on the last day LTG+FA (80mg/kg,p.o) + CBZ

Drugs and chemicals

Pentylenetetrazol (Sigma, Aldrich, USA) was dissolved in saline solution and was administered intraperitoneally (i.p.) to the mice on every alternate day. Lamotrigine (LTG) and carbamazepine (CBZ) (Prince Scientific, Telangana, India) were suspended in 0.5% carboxymethyl cellulose (CMC) and were administered i.p to the mice. Ferulic acid as treatment (Prince Scientific, Telangana, India) was suspended in CMC too, and was given orally to the mice on the last day at a dose of 40 and 80 mg/kg; doses were selected based on previous literature which showed FA neuroinflammatory action by decreasing the pro-inflammatory cytokines [13]. Enzyme-linked immunosorbent assay (ELISA) kits for IL-1β, TNF-α, and MMP-9 were purchased from ELK Biotechnology Ltd. (Wuhan, China).

Pretreatment with LTG and pentylenetetrazol-induced kindling

Drug-resistant epilepsy was induced in animals by LTG, pre-treatment (5 mg/kg) 45 minutes before pentylenetetrazol administration (30-40mg/kg, i.p.) [12] on every alternate day till the stable kindled state (three consecutive stages and five seizures) or till 19 injections of PTZ, based on whichever procedure was achieved prior in the mice (38 days). The seizure score was assessed using a modified Racine scale [14] described as Stage 0: no response; Stage 1: hyperactivity, restlessness, and vibrissae twitching; Stage 2: head nodding, head clonus, and myoclonic jerks; Stage 3: unilateral or bilateral limb clonus; Stage 4: forelimb clonic seizures; Stage 5: generalized tonic-clonic seizures with falling; Stage 6: hind limb extension. The mice were observed for 30 minutes in a plexiglass chamber after every PTZ injection.

Enzyme-linked immunosorbent assay

The brain tissues were carefully isolated on day 38 and stored at -80°C. Specific kits were used to record the concentration of pro-inflammatory cytokines (IL-1β and TNF-α) and MMP-9 in mice brain tissue. The experiments were performed as per the manufacturers’ instructions.

Preparation of mice hippocampal brain tissue and hematoxylin and eosin (H&E) staining

Brain tissue was collected by sacrificing animals with a high dose of ketamine and xylazine solutions. Hippocampal sections of the brain were isolated for histopathological examination and fixed in a 10% buffered formaldehyde solution. After that, an automated tissue processor (Leica Biosystems, Nußloch, Germany) was used to process the tissues fixed in formaldehyde. Each sample was embedded in the paraffin wax block. Tissue samples in these blocks were cut into 4 µm to 5 µm each section. These sections were then stained with H&E dye. The hippocampal region was examined under a light microscope and the ImageJ program (Bethesda, MD, USA) was used to assess any degenerative changes in the hippocampal neurons [15].

Statistical analysis

The data obtained are expressed as mean ± standard deviation (SD) and were analyzed for parametric or non-parametric tests with the Shapiro-Wilks normality test. Seizure score and biochemical data were analyzed with a one-way analysis of variance (ANOVA) test followed by Bonferroni post hoc analysis. The histopathological score data were analyzed with the Kruskal-Wallis test and the two groups were compared using the Mann-Whitney U test. The significance level was accepted as p<0.05. All statistical analyses were conducted using the Statistical Package for Social Sciences (SPSS) version 22 (IBM Corp., Armonk, NY, USA).

## Results

The effect of various treatments on the seizure score in the LTG/PTZ drug-resistant seizure model

**Figure 1:**
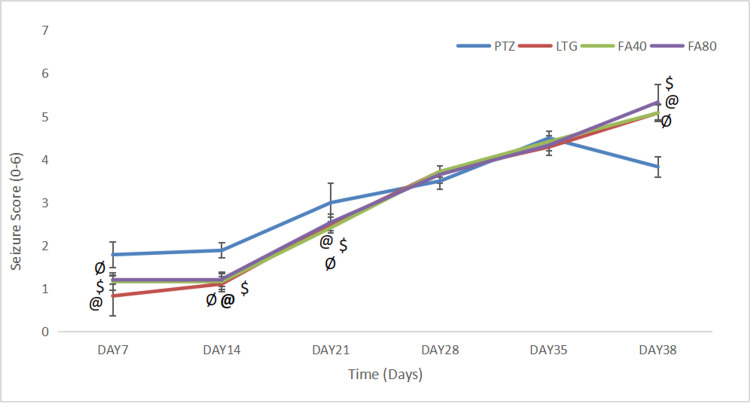
The seizure score in LTG/ PTZ drug-resistant seizure model in mice Data are expressed as mean ± SD. One-way ANOVA followed by Bonferroni post hoc analysis was applied. The sign ‘@’ indicates significance (p<0.05) between the PTZ and LTG+PTZ+CBZ treatment groups; ‘$’ indicates significance (p<0.05) between PTZ and LTG+FA (40mg/kg) + PTZ group+CBZ; ‘Ø’ indicates significance (p<0.05) between LTG+FA (80 mg/kg) + PTZ +CBZ and PTZ group; ‘#’ indicates significance (p<0.05) between LTG+PTZ+CBZ and LTG+FA (40mg/kg) + PTZ+CBZ group or LTG+FA (80 mg/kg) + PTZ +CBZ group. PTZ: Pentylenetetrazol, LTG: Lamotrigine; CBZ: Carbamazepine; FA: Ferulic acid, ANOVA: Analysis of variance, SD: Standard of deviation

In Group 1 (PTZ group), the mice showed a gradual increase in the seizure score from day 7 (1.79 ± 0.29) to day 38 (3.83 ± 0.24). Moreover, the subconvulsive LTG pre-treatment groups i.e., Group 2 (LTG+PTZ+CBZ), Group 3 (LTG+FA40mg/kg,p.o+PTZ+CBZ), and Group 4 (LTG+FA80mg/kg,p.o+PTZ+CBZ) showed a significant increase (p<0.05) in the seizure score from day 7 (0.83 ± 0.47) (1.17 ± 0.20) (1.21 ± 0.10 to) to day 38 (5.08 ± 0.19 ) (5.08 ± 0.20) (5.33 ± 0.41) when compared to the PTZ group. The two doses of FA showed no difference in the seizure score when compared to the LTG pre-treated group (Group 2). These effects on the seizure score are displayed in Figure 1.

The effect of various treatments on the latency to convulsive behavior after each PTZ administration

The mice in each group were observed for latency to develop seizures after PTZ administration. The LTG pre-treated groups i.e., Group 2 (LTG+PTZ+CBZ) treated group (201.37 ± 25.34s), Group 3 (LTG+FA40mg/kg,p.o+PTZ+CBZ) treated group (198.25 ± 12.05s), and Group 4 (LTG+FA80mg/kg,p.o+PTZ+CBZ) treated group (205.18±25.98s) significantly showed an increase (p<0.005) in the seizure latency to develop convulsive behavior after each PTZ injection as compared to Group 1 (PTZ treated group) at day 7 (89.33 ± 7.74s) (Table 2). However, no significant changes in seizure latency were observed at day 38 between the LTG pre-treated groups of Group 2 (LTG+PTZ+CBZ) treated group (97.08 ± 10.16s)], Group 3 (LTG+FA40mg/kg,p.o+PTZ+CBZ) treated group (105.42 ± 11.39s) and Group 4 (LTG+FA80mg/kg,p.o+PTZ+CBZ) treated group (97.08 ± 10.15) (Table 2).

**Table 2:** Latency to convulsive behavior after PTZ administration Data are expressed as mean ± SD. One-way ANOVA followed by Bonferroni post hoc analysis was applied. The sign ‘@’ indicates significance (p<0.05) between the PTZ and LTG+PTZ+CBZ treatment groups; ‘$’ indicates significance (p<0.05) between PTZ and LTG+FA(40mg/kg)+ PTZ group+CBZ; ‘Ø’ indicates significance (p<0.05) between LTG+FA(80 mg/kg)+PTZ +CBZ and PTZ group; ‘#’ indicates significance (p<0.05) between LTG+PTZ+CBZ and LTG+FA (40mg/kg)+PTZ+CBZ group or LTG+FA(80 mg/kg) + PTZ +CBZ group. PTZ: Pentylenetetrazol, LTG: Lamotrigine; CBZ: Carbamazepine; FA: Ferulic acid, ANOVA: Analysis of variance, SD: Standard of deviation

Time (days)	PTZ group (30-40mg/kg); n=6	LTG (5mg/kg) + PTZ +CBZ group; n=6	LTG+FA (40mg/kg) + PTZ + CBZ group; n=6	LTG+FA (80mg/kg) + PTZ+ CBZ group; n=6
7	89.33±7.74	201.37±25.34^@^	198.25±12.05^$^	205.18±25.98^Ø^
14	90.89±15.29	134.94±13.68^@^	132.11±11.02^$^	133.5±19.6^Ø^
21	95.63±4.99	94.04±7.66	97.46±8.27	99.75±12.69
28	20.42±18.46	102.83±18.65	125.11±15.31^#^	92.33±15.34^Ø^
35	108.58±6.67	99.16±10.06	98.33±11.98	99.04±9.91
38	162.08±23.04	97.08±10.16^@^	105.42±11.39^$^	97.08±10.15^Ø^

The effect of various treatments on the histopathological score (HPS) of the hippocampus in the drug-resistant seizure model

**Figure 2:**
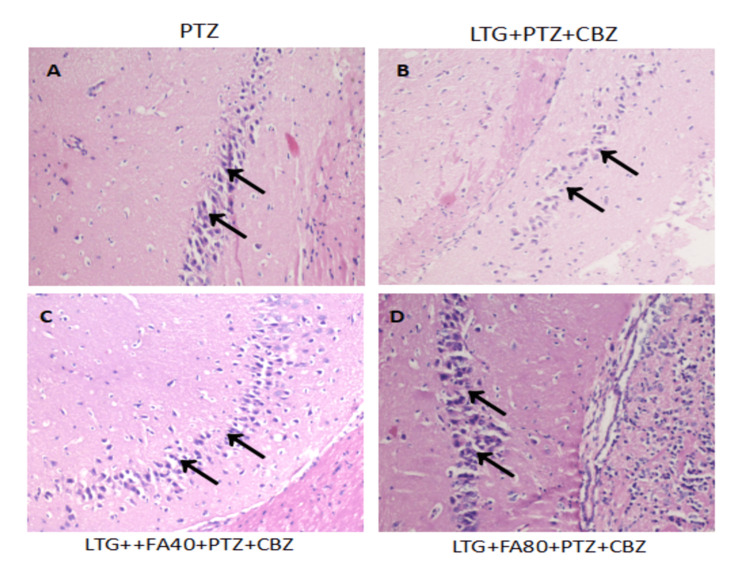
The effect of various treatments on the histopathology in LTG /PTZ drug-resistant seizure model in mice Microphotograph showing neuronal morphology in different experimental groups: (A) PTZ group showing injury to hippocampal neurons; (B) LTG+PTZ+CBZ  showing intense neuronal damage; (C) LTG+FA(40 mg/kg)+PTZ+CBZ group; and (D) LTG+FA(80 mg/kg)+PTZ+CBZ group showing diffused neuronal damage morphology. Please note that the densely stained nuclei of neurons are indicative of chromatin clumping (arrows) in H&E staining. Scale bar: 100 μm (20x). PTZ: Pentylenetetrazol, LTG: Lamotrigine; CBZ: Carbamazepine; FA: Ferulic acid, H&E: Hematoxylin and eosin

The hippocampal cells in the PTZ-treated groups showed intense neuronal injury, illustrating nuclear chromatin clumping, hypereosinophilia, and condensation of cytoplasm. The HPS in the PTZ group was 3.5 ± 0.54. Similarly, the neurons exhibited profound neuronal injury in the LTG+PTZ+CBZ treated group (HPS = 3.3 3 ± 0.51). Moreover, the neurons showed an exact level of neuronal damage in the LTG+FA40mg/kg,p.o+PTZ+CBZ treated groups. The HPS was 3.5 ± 0.54 in the LTG+FA40mg/kg,p.o+PTZ+CBZ group and 3.33 ± 0.51 in LTG+FA80mg/kg,p.o+PTZ+CBZ treated group. Both the doses of FA (40 mg/kg and 80 mg/kg) were comparable in the histopathological score as compared to the PTZ and LTG+PTZ+CBZ treated groups. The effect of the treatment on the HPS and a summary of the HPS are displayed in Figure 2 and Table 3, respectively.

**Table 3:** Degenerative changes and the histopathological score of mice hippocampus in LTG/ PTZ drug-resistant seizure model Data are represented as mean ± SD. Kruskal Wallis for all group data analysis and the Mann-Whitney rank test was applied for analysis between two groups. + mild, ++ moderate, +++ severe, - absent PTZ: Pentylenetetrazol, LTG: Lamotrigine; CBZ: Carbamazepine; FA: Ferulic acid, SD: Standard of deviation

Groups	Cytoplasmic vacuolation	Nuclear chromatin clumping	Hypereosinophilia and condensed cytoplasm	Fragmentation of cells	Histopathological score
PTZ	-	+++	++	-	3.5 ± 0.54
LTG + PTZ+CBZ	-	++	++	-	3.33 ± 0.51
LTG+FA (40mg/kg)+PTZ+CBZ	-	+++	+++	-	3.5 ± 0.54
LTG+FA (80mg/kg)+PTZ+CBZ	-	+++	+++	-	3.33 ± 0.51

The effect of various treatments on the brain cytokines levels of mice

Specific ELISA kits were used to estimate the levels of different cytokines (IL-1β, TNF-α, and MMP-9) in the mice's brains.

The Effect on IL-1β Level

**Figure 3:**
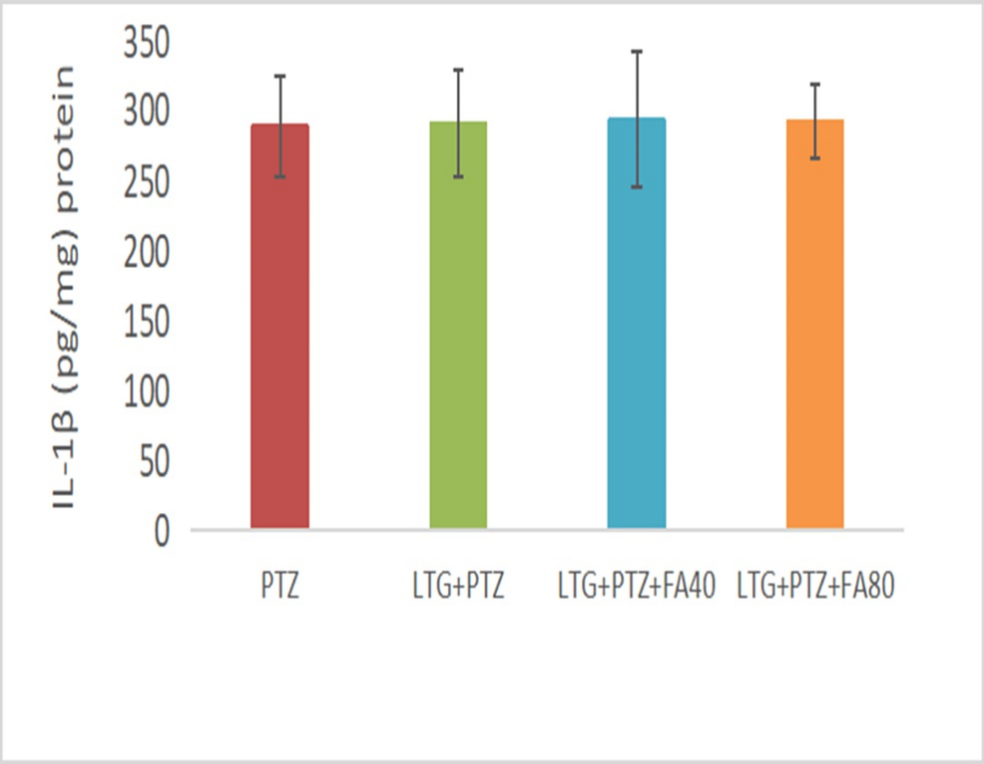
The effect of various treatments on the IL-1β level in LTG/PTZ drug-resistant seizure model in mice Data are expressed as mean ± SD. One-way ANOVA followed by Bonferroni post hoc analysis was applied. PTZ: Pentylenetetrazol, LTG: Lamotrigine; CBZ: Carbamazepine; FA: Ferulic acid, ANOVA: Analysis of variance, SD: Standard of deviation

The IL-1β levels (Figure 3) were observed to be upregulated in the PTZ (287.84 ± 36.15 pg/mg protein) group. Similarly, the LTG+PTZ+CBZ group showed increased IL-1β levels (290.02 ± 38.62 pg/mg), which were the same as compared to the PTZ group. In addition, LTG+PTZ+CBZ+FA (40 and 80 mg/kg) treatment showed comparable IL-1β levels (292.26 ± 48.47 pg/mg; 291.31 ±25.84 pg/mg) with the PTZ group.

The Effect on TNF-α Levels

**Figure 4:**
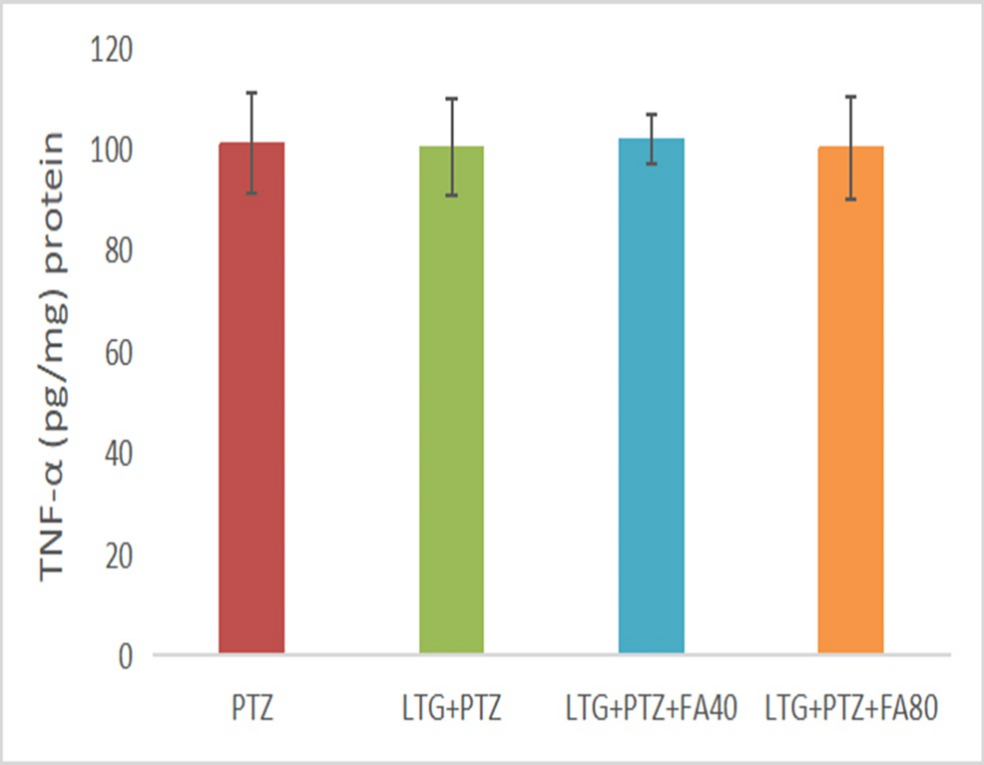
The effect of various treatments on the TNF-α level in LTG/PTZ drug-resistant seizure model in mice Data are expressed as mean ± SD. One-way ANOVA followed by Bonferroni post hoc analysis was applied. PTZ: Pentylenetetrazol, LTG: Lamotrigine; CBZ: Carbamazepine; FA: Ferulic acid, ANOVA: Analysis of variance, SD: Standard of deviation

The TNF-α levels (Figure 4) were found to be increased in the PTZ (100.88± 9.96 pg/mg protein) group. Similarly, the LTG+PTZ+CBZ group showed increased TNF-α levels (100.02 ± 9.40 pg/mg), which were the same as compared to the PTZ group. In addition, LTG+PTZ+CBZ+FA (40 and 80 mg/kg) treatment showed comparable TNF-α levels (101.82 ± 4.78 pg/mg; 100.13 ±10.14 pg/mg) with the PTZ group.

The Effect on MMP-9 Levels

**Figure 5:**
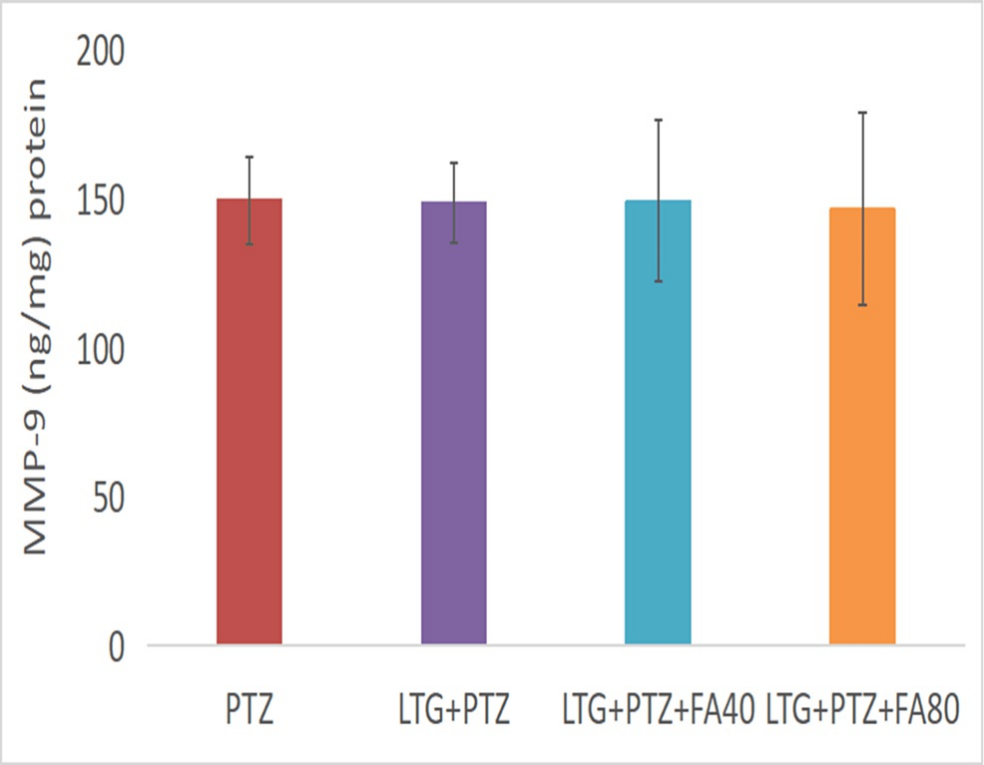
The effect of various treatments on the MMP-9 level in LTG/ PTZ drug-resistant seizure model in mice Data are expressed as mean ± SD. One-way ANOVA followed by Bonferroni post hoc analysis was applied. PTZ: Pentylenetetrazol, LTG: Lamotrigine; CBZ: Carbamazepine; FA: Ferulic acid, ANOVA: Analysis of variance, SD: Standard of deviation

The MMP-9 levels (Figure 5) were found to be increased in the PTZ (149.19 ± 14.65 ng/mg protein) group. Similarly, the LTG+PTZ+CBZ group showed increased MMP-9 levels (148.41 ± 13.40 ng/mg), which were the same as compared to the PTZ group. In addition, LTG+PTZ+CBZ+FA (40 and 80 mg/kg) treatment showed comparable MMP-9 levels (148.97± 26.99 ng/mg; 146.24 ±32.25ng/mg) with the PTZ group.

## Discussion

Drug-resistant epilepsy is one of the most challenging and burdensome diseases existing at present. About 30% of epileptic patients show refractory seizures due to intractable epilepsy. Such DRE patients are more prone to brain injuries and premature deaths. Thus, effective and targeted therapies are the need of the hour for DRE [16]. Natural polyphenols such as FA have shown neuroprotective actions in various animal models of brain injury and neurodegenerative disorders [17,18]. Besides, due to its antioxidant and hepatoprotective actions, it might be a potential therapeutic candidate over standard AEDs, which usually lead to hepatotoxicity and oxidative stress [19]. Altogether these findings led us to evaluate its potential in the animal model of DRE.

In the current study, LTG and PTZ were used to induce DRE, which manifests as recurrent seizures even after the administration of AEDs in Swiss albino mice. A similar finding had already been reported in a preclinical study with the same dose of LTG but different doses of PTZ [20] to establish DRE in both rats and mice. Ferulic acid (40 mg/kg and 80 mg/kg) did not ameliorate the seizure score or the latency in the animal model of DRE, which was induced by LTG and PTZ. However, past findings have reported the potent neuroprotective ability of FA in the pentylenetetrazol kindling model of epilepsy [21,22].

Due to the multivariate and peculiar nature of the disease, multiple hypotheses had been proposed to understand the mechanism of DRE that remains elusive [23]. Numerous studies have found a close correlation between neuroinflammation and the development of refractory epilepsy. In the central nervous system (CNS), after an injury or trauma, resident glia (astrocytes and microglia) release mediators such as pro-inflammatory cytokines and proteolytic enzymes that cause leaky BBB and finally lead to neuroinflammation [24].

Increased levels of pro-inflammatory cytokines like IL-1β, TNF-α, and MMP-9 are suggested to induce neuroinflammation in human and animal models of DRE [25]. In this study, IL-1β, TNF-α, and MMP-9 levels were significantly increased in mice treated with LTG and PTZ. Similar levels were demonstrated in mice treated with FA (40 and 80mg/kg) against the refractory model of epilepsy. However, a preclinical study on mice exposed to stress demonstrated contrasting results via the reduction in the pro-inflammatory and matrix metalloproteinases levels after the FA treatment [26]. This might be due to the acute treatment of FA given in our study and that too, orally. As per our knowledge, this would be the first study to explore the levels of IL-1β, TNF-α, and MMP-9 in the LTG/PTZ-induced drug-resistant seizure model. All these findings could lead to further evaluation of pharmacological interventions that reduce neuroinflammation and overcome uncontrollable seizure activity.

Due to the antagonistic action of PTZ at the γ-aminobutyric acid (GABAa) receptor, it leads to intense neuronal injury in the brain of mice [27]. Moreover, the treatment of LTG-kindled mice with two doses of FA (40 and 80mg/kg) and CBZ was not able to mitigate the damage to the hippocampal neurons in mice. One major explanation could be the similar mechanism of action shared between LTG and CBZ that leads to cross-tolerance [28]. Our results were in contrast with a previous study where FA has been reported to preserve neuronal integrity [29] in an animal model of pentylenetetrazol kindling. 

## Conclusions

The current study demonstrated that FA did not show neuroprotection against LTG/PTZ-induced kindling models of DRE in mice. No changes in the pro-inflammatory cytokines and hippocampal neuronal injury suggest that FA does not alter inflammation when given orally for the acute time frame (single administration) in an animal model of drug-refractory epilepsy. Meanwhile, further experimental models of DRE should be explored, where FA could be tested chronically to properly evaluate its efficacy as a neuroprotectant.
